# Diverse and Composite Roles of miRNA in Non-Neuronal Cells and Neuronal Synapses in Alzheimer’s Disease

**DOI:** 10.3390/biom12101505

**Published:** 2022-10-17

**Authors:** Xinrong Li, Shih-Chi Chen, Jacque Pak Kan Ip

**Affiliations:** 1 Department of Biomedical Engineering, City University of Hong Kong, Hong Kong 999077, China; 2 Hong Kong Centre for Cerebro-Cardiovascular Health Engineering (CoCHE), Hong Kong Science Park, Hong Kong 999077, China; 3 Department of Mechanical and Automation Engineering, The Chinese University of Hong Kong, Hong Kong 999077, China; 4 School of Biomedical Sciences, The Chinese University of Hong Kong, Hong Kong 999077, China

**Keywords:** Alzheimer’s disease, microRNA, astrocyte, microglia, cerebrovasculature, synapse

## Abstract

Neurons interact with astrocytes, microglia, and vascular cells. These interactions become unbalanced in disease states, resulting in damage to neurons and synapses, and contributing to cognitive impairment. Importantly, synaptic loss and synaptic dysfunction have been considered for years as a main pathological factor of cognitive impairment in Alzheimer’s disease (AD). Recently, miRNAs have emerged as essential regulators of physiological and pathological processes in the brain. Focusing on the role of miRNAs in regulating synaptic functions, as well as different cell types in the brain, offers opportunities for the early prevention, diagnosis, and potential treatment of AD-related cognitive impairment. Here, we review the recent research conducted on miRNAs regulating astrocytes, microglia, cerebrovasculature, and synaptic functions in the context of AD-related cognitive impairment. We also review potential miRNA-related biomarkers and therapeutics, as well as emerging imaging technologies relevant for AD research.

## 1. Introduction

MicroRNAs (miRNAs) are a class of non-coding RNAs (about 21 nucleotides) that play a crucial role in regulating gene expression in eukaryotes and are highly conserved [1]. miRNAs are transcribed from DNA sequences by RNA polymerase II (Pol II) into primary miRNAs (pri-miRNA) with local hairpin structures embedded with miRNA sequences, then precursor miRNAs, and eventually mature miRNAs [1]. The miRNA-induced silencing complex (miRISC) is a core component of the miRNA-mediated gene silencing process, and its primary function is to discriminate and repress miRNA-targeted mRNAs [2]. The centerpiece structure of miRISC is the GW182 protein and the Argonaute (Ago) protein loaded with mature miRNA [3]. GW182 has contributed to miRNA-mediated target-gene-expression silencing. The functional silencing of target gene expression by the Ago–miRNA interaction is disturbed when miRNAs are not entirely complementary to their target mRNAs [4]. GW182 binds to and silences target mRNAs independent of Ago to ensure translational repression, decapping, deadenylation, and mRNA degradation [3]. Interestingly, the strength of the GW182–Argonaute interaction is affected by whether the guide RNA is loaded on the Ago [5]. Multiple reports have revealed that miRNAs can alternatively target the miRNA-recognition elements (MREs) in protein-coding sequence (CDS) regions in an Ago-dependent but GW182-independent manner in order to suppress translation by inducing transient ribosomal arrest rather than mRNA destabilization, resulting in gene silencing [6]. A recent study on Drosophila suggests that the middle region domain of GW182 significantly contributes to circular RNA (circRNA) degradation. GW182 may regulate at least a subset of circRNA degradation in an Ago-slicer or P-body-independent manner [7]. In this review, we discuss the diverse roles of miRNA in non-neuronal cells and neuronal synapses in AD.

## 2. Role of miRNAs in Regulating Astrocytes and Microglia in AD

**Figure 1:**
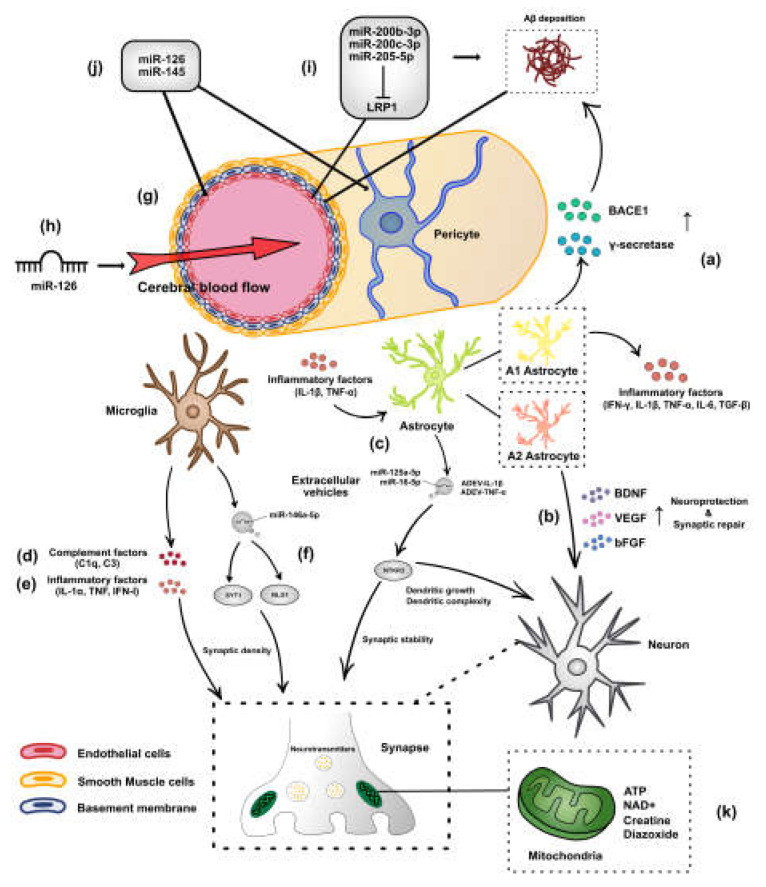
Interconnection of synapses and the cerebrovascular system. (**a**) A1 astrocytes cause a high production of Aβ by producing inflammatory factors, including IFN γ, IL-1β, TNF-α, IL-6, TGF-β, and massive amyloid precursor protein-cleaving enzymes, such as β-secretase (BACE1) and γ-secretase. (**b**) A2 astrocytes release neurotrophic factors, such BDNF, VEGF, and bFGF, which contribute to synaptic repair and neuroprotection. (**c**) Astrocytes secrete inflammatory factors, such as IL-1β and TNFα, to stimulate the production of ADEVs, carrying miR-125a-p and miR-16-5p to target NTKR3 to regulate synaptic stability. (**d**) Microglia alter the synaptic phenotypes in the hippocampal neuron by releasing a series of complement factors (e.g., C1q and C3), (**e**) inflammatory factors (e.g., IL-1α, TNF, and type-I IFN), and (**f**) extracellular vesicles (EVs) to deliver miR-146a-5p to target SYT1 and NLG1 to regulate synaptic density. (**g**) The cerebrovascular system is composed of endothelial cells, smooth muscle cells (SMCs), pericytes, perivascular immune cells (e.g., microglia), and surrounding astrocytes. Abnormal cerebral blood flow leads to a greater tendency for Aβ to be deposited within the basement membrane of blood vessels. (**h**) miR-126 in the endothelial cells participate in the regulation of cerebral blood flow. (**i**) miR-200b-3p, miR-200c-3p, and miR-205-5p considerably reduced low-density LRP1 in microvascular endothelial cells impairing Aβ clearance. (**j**) miR-126 and miR-145, specifically in brain capillaries, elevate pericyte coverage as well as promote endothelial cell activation. (**k**) Providing neurons with mitochondria-related energy chemicals, such as adenosine triphosphate (ATP), nicotinamide adenine dinucleotide (NAD+), creatine, or diazoxide are efficacious treatments that can effectively protect neurons from metabolic and Aβ-toxicity damage.

Astrocytes are the most abundant cell type in the brain [8]. The roles of astrocytes in AD depend on the neuronal environment. Reactive astrocytes are classified as A1 and A2. A1 astrocytes promote neuroinflammation by producing massive amyloid precursor protein-cleaving enzymes, β-secretase (BACE1) and γ-secretase, causing a high production of Aβ and secreting inflammatory factors, such as IFN γ, IL-1β, TNF-α, IL–6, and TGF-β [9] (Figure 1a). Conversely, A2 astrocytes can upregulate neurotrophic factors, such as brain-derived neurotrophic factor (BDNF), vascular endothelial growth factor (VEGF), and basic fibroblast growth factor (bFGF), contributing to neuroprotection and synaptic repair [10] (Figure 1b). Astrocytes remove cellular debris by releasing extracellular vesicles (EVs), a mechanism for the homeostatic regulation of the neuronal function [11,12]. EVs are membrane vesicles (MVs) produced by the germination of plasma membranes (ectosomes) or by the cytosolic action of multivesicular bodies (exosomes). Individual EVs carrying specific molecular messages in the form of RNA, proteins, and lipids play a role in transmitting signals between cells [13]. Astrocyte-derived small extracellular vesicles (sEVs) reduce hippocampal dendritic complexity by upregulating endogenous miR-26a-5p levels and potentially regulating dendritic spines and synaptic transmission as well [14]. Astrocyte-derived extracellular vesicles (ADEV) are a novel astrocyte–neuron communication mediator that has recently been discovered [11]. ADEVs are released in response to IL-1β (ADEV-IL-1β) and TNFα (ADEV-TNFα) enriched with miR-125a-5p and miR-16-5p, targeting NTKR3 and its downstream effector Bcl2 to regulate dendritic growth, dendritic complexity, and synaptic stability [15] (Figure 1c).

Microglia interact with neurons, astrocytes, and oligodendrocytes in the brain, and mediate synaptic dysfunction or loss in AD through multiple pathways. The activation of microglia is closely related to the activation of the complement system [16]. The activation of complement-mediated mechanisms (e.g., C1q and C3) has been demonstrated to directly mediate synaptic loss and dysfunction in AD mice [17,18] (Figure 1d). C1q and C3 levels are parallel to Aβ deposition in AD mice [19]. Rescuing cognitive impairment in AD induced by Aβ-mediated synaptic loss by reducing C1q and C3 levels is a potential therapeutic avenue [20,21,22]. Microglia destabilize its homeostasis to an inflammatory state and release a series of inflammatory factors, such as IL-1α, TNF, and IFN-1, leading to synaptic loss, neuronal defects, and alterations in learning and memory [23,24] (Figure 1e). AD-related risk genes expressed by the microglia (e.g., TREM2 and ApoE) are demonstrated to mediate synaptic loss [25]. The microglia are able to modulate the expression of dendritic spines and synapse-associated proteins by releasing EVs to deliver miR-146a-5p that are not originally present in the neurons, to alter the number of dendritic spines and synaptic function in hippocampal neurons by repressing the translation of presynaptic synaptotagmin1 (Syt1) and postsynaptic neuroligin1 (Nlg1) [26] (Figure 1f). miR-146a prevents synaptic loss in AD through the alteration of microglia polarization, a phagocytic phenotype that reduces the secretion of inflammatory factors and protects neurons in AD [27].

## 3. Role of miRNAs in Regulating the Cerebrovascular System in AD

The cerebrovascular system is composed of a complex community of cells that interact with each other to enable the proper functioning of material circulation, metabolism, and permeability properties within the brain, including endothelial cells, smooth muscle cells, pericytes, perivascular immune cells (e.g., microglia), and surrounding astrocytes (Figure 1g). It is essential for maintaining brain health. Cerebrovascular disease (CVD) and AD share several risk factors [28], such as APOE ε4 [29], midlife hypertension, and hypercholesterolemia [30,31]. Vascular risk factors are predictive of the progression of AD and the conversion of mild cognitive impairment (MCI) to AD [32,33]. One of the most prominent implications is the abnormalities of cerebral blood-flow autoregulation and impaired removal of metabolic waste and neurotoxins from the brain. The paravalvular interstices around the cerebral vessels provide a low-resistance pathway for the convective clearance of solutes in the brain, with major contributions from the lymphatic and glymphatic systems (water channels represented by AQP4) [34,35]. Interstitial fluid drains from the brain along with the perivascular space and basement membrane [36]. Mouse models of cerebral amyloid angiopathy display abnormal cerebral blood flow and a greater tendency for Aβ to be deposited within the basement membrane of blood vessels [37].

The cerebrovascular system governs cerebral blood flow, the delivery of nutrients to the brain, the filtering of the blood–brain barrier (BBB) for brain-entering substances, and angiogenesis. Emerging evidence indicates that these factors are closely related to AD [38,39,40]. A novel technique, vessel isolation and nuclei extraction for sequencing (VINE-seq), has been developed to investigate genome-wide association studies (GWASs) in the vascular system of cortical and hippocampal samples obtained from Alzheimer’s patients. Surprisingly, the results show that up to 30 of the top 45 genes linked to AD are expressed in the vascular system of the human brain [41]. Cells of the human brain vasculature predominantly include endothelial cells, smooth muscle cells, pericytes, astrocytes, macrophages, T cells, and perivascular and meningeal fibroblasts.

miRNAs intervene in cardiovascular diseases by participating in the pathogenic mechanism of the disease. A decrease in cerebral blood flow to the striatum, hippocampus, or thalamus in the deep part of the brain triggers inflammation resulting in organ damage, causing the impairment of cognitive and memory functions [42]. The deletion of miR-126 in endothelial cells decreases the cerebral blood flow in mice (Figure 1h) and activates the astrocytes and microglia, thereby increasing inflammation as well as triggering the impairment of water channels and the lymphatic system, lower synaptic plasticity, and dendritic spine density [43]. Changes in miRNA profiles were detected while improving cerebral blood flow in a stroke model with therapies, indicating the possible involvement of miRNAs in the mechanism of improving cerebrovascular and neurological functions [44]. The role of miRNAs in vascular cognitive impairment progression warrants investigation.

Cerebrovascular miRNA markers corresponding to post-transcriptional regulation can indicate the stage of AD pathogenesis [45]. In the total brain vessel segments of 3xTg-AD mice, let-7g, miR-1944 (in males), miR-133a, and miR-2140 (in females) were significantly downregulated upon the transition from the young stage (1–2 months old) to cognitive impairment (CI). miR-99a in males was downregulated during the period of CI to extracellular Aβ formation. From the pre-AD (3–5 months old) to AD (6–15 months old) phase, let-7d, let-7i, miR-23a, miR-34b-3p, miR-99a, miR-126-3p, miR-132, miR-150, miR-1151-5p, miR-181a (in males), and miR-150, miR-539 (in females) were detected as being markedly altered in expression [45]. miR-200b-3p, miR-200c-3p, and miR-205-5p considerably reduced low-density lipoprotein receptor-related protein 1 (LRP1) in microvascular endothelial cells (MVECs) [46]. LRP1 impaired Aβ clearance [47,48,49], allowing Aβ peptides but not Aβ plaques, soluble and insoluble Aβ40, and Aβ42 to accumulate in the cortex, triggering increased microglia and astrocytes to induce neuroinflammation and to impair spatial memory in mice (Figure 1i). This process was induced by copper [46]. A high expression of miR-126 and miR-145, specifically in the brain capillaries, elevated pericyte coverage and promoted endothelial cell activation, resulting in an increased clearance of Aβ oligomers through the perivascular route [50] (Figure 1j). miR-181a is suspected to be involved in hippocampal memory formation [51,52]. Amyloid precursor protein (APP) is an important factor impacting synaptogenesis and neuronal health, and its presence is bidirectional and not just harmful as a source of Aβ [53]. miRNA is essential in the homeostatic regulation of APP dynamics in vivo [54]. miR-181a negatively targets forkhead box protein O1 (FOXO1), decreasing amyloid plaque deposition in the hippocampus and cortex, mitigating pericyte loss and BBB disruption in APP/PS1 mice, and ameliorating cognitive impairment in mice [55]. As the most abundant miRNA in the brain tissue, miR-124 is involved in the pathological process of age-related ischemic encephalopathy [56,57,58,59]. Paradoxically, miR-124 demonstrates beneficial aspects in ischemic encephalopathy by regulating autophagy, neuroinflammation, oxidative stress, Aβ deposition, and tau protein hyperphosphorylation [59,60,61], while in AD, it may adversely affect synaptic plasticity via the miR-124/PTPN1 pathway, through neural differentiation by mediating apoptosis [62,63] (Table 1). A thorough understanding of the role of miR-124 in ischemic encephalopathy and AD could help develop new avenues for the exploration of ischemic encephalopathy and AD prevention or treatment therapies.

## 4. miRNAs Are Associated with AD-Related Synaptic Impairment

Dendrites and dendritic spines are functionally critical structures in cognitive processes, such as learning and memory, and dysfunction and morphological changes in dendritic spines are essential characteristics of AD [73]. Pre-AD-related cognitive impairment is correlated with the progressive loss of synapses and neurons [74]. Synaptic density is believed to be a better predictor of a person’s cognitive performance than the loss of gray-matter volume in the brain, and this correlation also extends to the mild and pre-AD stages [75]. Synapses interconnect neurons via electrical and chemical signaling (e.g., glutamate and GABA). Animal studies have indicated that dendritic spine morphological changes preceded synaptic loss [76], and showed an increased proportion of abnormal spine morphology. The shortening and widening of the neck of the dendritic spine alter the electrical compartmentalization of the spines, resulting in reduced postsynaptic potentials in the head of the spine, long-term potentiation (LTP) enhancement, and impaired spatial memory [77]. In recent years, a series of publications have demonstrated the regulation of miRNAs on synapses, including synaptic plasticity, synapse formation, synapse morphology, and synapse quantity. The overexpression of miR-34a in AD inhibits presynaptic and postsynaptic proteins, such as VAMP2, SYT1HCN, NR2A, GluR1, and SHANK3 [78]; it impairs rapid synaptic-vesicle endocytosis, fast calcium-triggered synaptic vesicle exocytosis, synaptic input integration, and synaptic plasticity [79]. A meta-analysis of the gene expression data showed that miR-129 was substantially upregulated in the hippocampus of scopolamine-induced AD rats and was concentrated in the synapses with a high expression. miR-129 has over 600 target genes in the hippocampus, four of which are considered to play a function in synaptic plasticity, LTP, and long-term depression (LTD): CAMK2D, CAMK4, PPP3CA, and PRKCB, which encode CaMKII, CaMKIV, CaN, and PKC, respectively [67]. This result echoes another meta-analysis predicting that miR-30a-5p targets CAMK2B, CAMK3, MAPK1, PPP3CB, and RSP6KA2 in the LTP in AD [80]. Here, we summarized several miRNAs related to synapses in AD that were exploited by bioinformatic tools or experimental phases in recent years (Table 1).

### 4.1. miRNAs Involved in Aβ-Mediated AD Synaptic Dysfunction

Aβ interacts directly or indirectly with various synaptic proteins to disrupt synaptic function, such as AMPAR, NMDAR, mGluR5, PRPC, RAGE, and ephrin type-B receptor 2 (EPHB2).

miR-30b: miR-30b, which is highly analogous to miR-30a, has the NF-κB p65 subunit, which binds to miR-30b and is regulated by upstream Aβ42 to promote its upregulation in the brains of AD patients and APP transgenic PSD mice. Nevertheless, the specific mechanism of how upstream Aβ42 regulates miR-30b is unclear, and the expression of the enzymes that produce and synthesize Aβ does not change. miR-30b overexpression impairs synaptic integrity and cognitive function. Its target genes are EPHB2, sirtuin1 (SIRT1), and glutamate ionotropic receptor AMPA-type subunit 2 (GluA2), which are essential for maintaining synaptic integrity, exhibiting the downregulated expression in AD. The knockdown of miR-30b in TG mice rescued dendritic spine density, GluA1, GluN2B, and PSD95 expression levels in the hippocampal neurons, presumably mediated by EPHB2 and SIRT1 [71].

miR-181a: miR-181a acts as a negative regulator of synaptic plasticity and a positive regulator of memory. miR-181a mediates Aβ-induced synaptotoxicity in the mouse hippocampus. miR-181a and its target protein, GluA2, are reduced and increased, respectively, during the process of learning. In this way, miR-181a effectively reversed Aβ oligomers-induced impairments in synaptic plasticity and memory deficits in 3xTg-AD mice. Yet, PSD95, SYP, and GluA1 presented no significant differences [51]. In accession to GluA2, miR-181a has been proven to target CREB1 and PRKAA1, which are transcription factors and proteins that function in learning and memory, respectively [51,52].

miR-132: the significance of miR-132 in neural development and the regulation of synaptic function has been widely studied [81]. miR-132 acts as a mediator of inflammation [82,83]. An enriched environment enhances hippocampal LTP by upregulating miR-132 and reducing histone deacetylase (HDAC) signaling, thereby counteracting the synaptotoxicity of Aβ and acting as a preventive factor against hippocampal damage. This implies that the accumulation of Aβ in the brain can be reduced by prolonged stimulation with novelty, which may prevent AD [68].

miR-34: the expression of miR-34a plays a predictive function in Aβ-mediated AD, as its expression is upregulated before the aggregation of Aβ [84]. The inhibition of miR-34c rescued Aβ-induced AD-related spine morphology and synaptic integrity and memory damage by targeting SYT1 through the ROS-JNK-p53 pathway. However, its upstream regulatory signaling pathways and downstream molecular mechanisms in AD are unclear [72].

### 4.2. Regulation of Synaptic Function by miRNA-Mediated Tauopathies

Tau is a microtubule-associated protein. In the human genome, it is derived from the selective splicing of microtubule-associated protein tau (MAPT) mRNA [85]. Tau maintains the assembly and structural maintenance of microtubule structures through the binding of microtubule structures in normal physiological conditions [86,87]. Tau is abundantly expressed in central nervous system neurons and weakly expressed in astrocytes and oligodendrocytes. Structural defects in tau proteins are believed to be one of the cardinal pathogeneses of AD [88,89].

Bilen et al. [90] first raised the prospective role of miRNAs in tauopathies in a Drosophila study. They observed that the loss of the protein R3D1/loquacious blocked miRNA processing, preventing miRNA maturation, and significantly increased tau-mediated neuronal death [90]. miRNAs regulate tau phosphorylation in two principal ways: the first is direct binding to the 3’UTR of the mRNAs of protein kinases and phosphatases. The second is indirect regulation by modulating the factors involved in activating protein kinases and phosphatases. Smith et al. showed that miR-124, miR-9, miR-132, and miR-137 caused the misplacement of tau exon 10, leading to an imbalance in the 4R:3R-tau ratio in neuronal cells and resulting in disease [91]. Phosphorylation is the most frequent post-transcriptional modification of tau, usually occurring in the serine (Ser), threonine (Thr), and tyrosine (Tyr) residues of the protein [92].

miR-132: miR-132 acts as a positive regulator during postnatal brain development. miR-132 directly targets the polypyrimidine tract-binding protein 2 (PTBP2) in the neurons to aberrantly splice tau exon 10, leading to an imbalance in the ratio of three-repeat (3R) and four-repeat (4R) tau isoforms [91]. miR-132 prevents microtubule dynamics and neurofibrillary tangle (NFT) accumulation to enhance LTP in the P301S tau transgenic mice by directly targeting the RNA-binding protein RNA Binding Fox-1 Homolog 1 (RBFOX1) to degrade tau mRNA, acetylation via EP300, cleavage via calpain 2 and caspases 3/7, and tau kinase glycogen synthase kinase 3β (GSK3β), which hyperphosphorylates tau [69] to disrupt microtubule dynamics and NFT accumulation [93]. In addition to this, miR-132 can target glycosyltransferase-like domain containing 1 (GTDC-1), indirectly inhibiting the tau kinase cyclin-dependent kinase 5 (CDK5) through the NOS1 signaling pathway [94].

miR-135a-5p: miR-135a-5p displays a Foxd3-mediated tau-dependent expression decline in AD mice. In MAPT knockout neurons, tau overexpression was able to induce a loss of miR-135a-5p expression, leading to reduced LTP and basal transmission in CA3-CA1 projections, as well as reduced hippocampal pyramidal neuronal dendritic complexity and spine maturation [64]. Rho-Associated Coiled-Coil Containing Protein Kinase 2 (ROCK2) is one of six potential major regulators of synaptic and axonal degeneration in vivo [95]. ROCK2 is the direct target of miR-135a-5p. The loss of miR-135a-5p expression results in aberrant ROCK2 activation and the subsequent deregulation of the cellular cytoskeleton via phosphorylation of ADD1 at Ser726. The interaction of ROCK2 with its downstream target ADD1 promotes synaptic dysfunction. [64]. Synaptic density, synaptic transmission, and hippocampal LTP are considerably impaired in ROCK2 knockout mice [96]. The overexpression of ROCK2 also impairs dendritic morphology and LTP and basal transmission [64].

miR-101b: previous studies have revealed that HDAC is closely associated with excessive Aβ production [68,97]. Liu et al. identified that HDAC2 is also involved in the hyperphosphorylation of tau [98]. Tau is one of the cytoplasmic substrates of HDAC. HDAC inhibitors, such as Scriptaid, M344, BML281, and SAHA, can increase the level of acetylated tau, leading to the activation of the tau pathology [99]. 5′-adenosine monophosphate (AMP)-activated protein kinase (AMPK) reduces Tau phosphorylation and improves brain function in C57 mice [100]. The AMPK activity is significantly reduced in aging C57 mice and 3 × Tg AD mice. It inhibits GSK3β activity in HEK293 tau cells transfected by the AMPK plasmid [100]. The ectopic expression of HDAC2 promotes hyperphosphorylation and the aggregation of tau, and scrambles the combination of the hepatocyte nuclear factor 4α (HNF-4A) transcription factor with the miR-101b promoter. This results in the upregulation of AMPK, the target protein of miR-101b, a loss of spine density, and memory deficits in AD mice [98].

miR-9: miR-9 has been reported to play a vital role in neurodevelopment, neurogenesis, neuronal morphology, and dendrite development [101,102]. SIRT1 is a histone deacetylase abundantly expressed in the brain and is closely associated with transcriptional silencing, aging, and inflammation [103]. SIRT1 serves as a target of miR-9-5p, which is also one of the critical enzymes for removing acetyl groups from tau. Its deficiency causes synaptic loss and premature death in tauP301S transgenic mice. In contrast, the stereotaxic delivery of the adeno-associated virus that encodes SIRT1 into the hippocampus reduces acetylated Lys174 (K174) of tau [70]. UBE4B, an E3/E4 ubiquitin ligase, functions as a target gene for miR-9a, and its overexpression enhances the ubiquitination and degradation of tau. In a Drosophila tau overexpression model, UBE4B alleviated eye neurodegeneration and synaptic bouton defects [104]. However, the relationship between UEB4B and synaptic function is unclear.

miR-138: in the previous subsection, we briefly described the significance of SIRT1 in synapse and neural development. In addition to miR-9, miR-138 targets SIRT1 to regulate tau pathology. Interestingly, the in vitro overexpression of miR-138 in cultured cells was associated with increased Aβ production and tau phosphorylation, whereas in vivo experiments also showed an increased production of soluble Aβ42 and significantly reduced tau expression in the hippocampus and frontal cortex, but there was no significant effect on phosphorylation (PHF1, AT8, and S422 phosphorylation epitopes) [105]. The effects of miR-138 on the synapses were primarily manifested by increased postsynaptic marker PSD95, and decreased presynaptic marker synaptophysin and synaptic density [65,105]. In terms of cognition, the upregulation of miR-138 induced alterations in learning and memory, as well as anxiety-like behavior in mice [65].

## 5. Mitochondria as a Potential Therapeutic Target for AD-Related Cognitive Disorders

Neuronal energetics is related to neurotransmission. Most of the energy employed by neurons is used for synaptic signaling [106]. Hippocampal pyramidal neurons have the highest energy demand of all neurons in the brain [107]. Therefore, when the metabolic and energetic demands of hippocampal neurons are not satisfied, they are more at risk for cognitive impairment. In the early stages of AD development and aging, cellular metabolism becomes disturbed, causing a disturbance in the dynamic balance between glutamatergic and GABAergic signals released by the neurons and synapses [108]. By providing neurons with energy chemicals, such as adenosine triphosphate (ATP) [109], nicotinamide adenine dinucleotide (NAD+) [110], creatine [111], or the mitochondrial potassium channel-opener diazoxide [112], they can effectively protect the neurons from metabolic and Aβ toxicity damage and are expected to improve cognitive impairment (Figure 1k).

The mitochondria provide 90% of cellular energy as ATP. A study of brain tissue obtained from AD patients found region-specific changes in the synaptic mitochondria in AD [113]. Mitochondrial transport to the presynaptic terminal or synaptic mitochondrial dynamics may be altered in AD with abnormal mitochondrial morphology and the presence of multivesicular bodies in the synapse [113]. miR-455-3p dysregulation in the neuronal mitochondria is closely related to synaptic activity [114]. Mitochondrial miRNAs are emerging targets in AD and synaptic research [115]. Mitochondrial miRNAs have the potential for the development of biomarkers and targeted therapies for AD. However, research regarding the effects of mitochondrial miRNAs on specific mitochondrial functions is limited.

## 6. miRNAs as Potential Therapeutics or Biomarkers of AD

The FDA approved the first miRNA-based therapy for hereditary transthyretin amyloidosis in 2018 [116]. Biotechnology companies, such as Miragen, Synlogic, and Regulus therapeutics, are focused on developing miRNA-related medication. Nevertheless, there are no FDA-approved miRNA therapies for AD in current clinical use, and it is still in the preclinical phase.

The application of miRNA therapies in AD remains behind other disease areas, and more preclinical work is in demand. How to penetrate the BBB to target the brain to deliver miRNA supplements or inhibitors is a pivotal challenge that needs to be addressed. miRNA medications are selected for in situ injection instead of intravenous administration in cancer therapies in order to strengthen the target specificity and minimize side effects [117]. Nonetheless, the greatest obstacle to oligonucleotide-based miRNA-targeted treatments for AD is, at present, navigating the medicine to cross the BBB; only fat-soluble small molecules below 400 daltons can pass the BBB. Researchers have shown in rats that antimiR-134 can be selectively transfected into striatal neurons for the treatment of Parkinson’s disease by intracerebroventricular injection of the magnetic particle NeuroMag^®^ complex plasmid into the lateral ventricles of the pars striatum, and the expression of miR-134 decreased by 0.35 fold [118].

Individual miRNAs can target multiple mRNAs, although their effects might be contradictory [119], whereas multiple miRNAs can also simultaneously target one mRNA [120]. It has been proposed that MG-6267, a dual inhibitor of AChE and miR-15b biogenesis, is able to address the multifactorial properties of AD as a therapeutic strategy. Preliminary predictions suggest that MG-6267 has a high CNS penetration [121].

In human patients, miRNA cannot be extracted from brain tissues as in animals, and samples are mainly derived from either peripheral blood or cerebrospinal fluid, especially in the exosomes. A clinical study predicted miRNA biomarkers in the peripheral blood serum exosomes of AD patients by high-throughput next-generation sequencing (NGS) and conducted random forest analyses with patients’ clinical, medical, and cognitive assessments and amyloid neuroimaging with positron emission tomography. A total of 16 miRNAs (hsa-miR-101-3p, hsa-miR-106a-5p, hsa-miR-106b-5p, hsa-miR-1306-5p, hsa-miR-143-3p, hsa-miR-15a-5p, hsa-miR-15b-3p, hsa-miR-18b-5p, hsa-miR-20a-5p, hsa-miR-30a-5p, hsa-miR-335-5p, hsa-miR-342-3p, hsa-miR-361-5p, hsa-miR-424-5p, hsa-miR-582-5p, and hsa-miR-93-5p) were obtained that were responsive to patients’ AD progression and had potential as peripheral screening tools [122].

## 7. Emerging Imaging Techniques for AD Research and Potential Applications in Diagnosis

### 7.1. Two-Photon Microscopy

Using two-photon imaging techniques, researchers have revealed in vivo in APP/PS1 mice that deficits in spine maintenance and plasticity occur in specific brain regions [123,124,125]. Reactive astrocytes can be observed in the brains of patients with mild cognitive impairment in the early stages of AD, as well as in APP/PS1 mice, and precede the appearance of amyloid plaques [126]. In vitro astrocyte studies failed to fulfill the demands of exploring the relationship between astrocytes, neurons, and the vascular system. Employing genetically encoded calcium indicators (GECIs), such as GCaMP, provided a stable and specific fluorescent indicator for studying calcium signaling and synaptic function in astrocytes [127]. Fluorescent probes for Aβ proteins [128], molecular probes for p-tau proteins [129], metal-ion fluorescent probes [130], two-photon monoamine oxidase (MAO) probes [131], reactive astrocyte probes [126], and miRNA probes [132] have been developed for two-photon imaging. Fluorescent probes not only serve as a localization tool for biomarkers, but also serve as potential targeted therapeutics based on the pharmaceutical molecules they carry. For example, curcumin has anti-inflammatory and antioxidant properties. Curcumin two-photon probes capable of targeting Aβ is being considered as a theranostic agent due to its imaging and potential therapeutic functions [133]. Although there are still technical challenges to be overcome, including low-fluorescence quantum yields, an inability to detect soluble Aβ, and difficulty in penetrating the BBB, this finding provides evidence for visualizing the effects of targeted therapy [133]. Another limitation at present is that two-photon in vivo imaging is limited to a 450 μm depth from the brain surface and requires replacing part of the skull with a cranial window to track fluorescently labeled neurons [134]. The synaptic imaging of the hippocampus requires more invasive micro-endoscopy or removing the overlying cortical tissue [135].

### 7.2. Optical Coherence Tomography (OCT)

Optical coherence tomography is an imaging technique that uses low-coherence light to capture two- and three-dimensional images from within the biological tissue with micron-level resolution. In the field of medicine, it is commonly applied in ophthalmology, cardiology, endovascular applications, and oncology. In the field of cardiology, researchers have used OCT to scan patients’ coronary arteries as well as to measure circulating miRNA levels from patients’ serum samples, with the finding that a decrease in miR-24 is strongly associated with coronary neointimal hyperplasia [136,137]. A study in the atherosclerotic rabbit model found that miR-let-7b regulates macrophages by targeting the TGF-βR1 signaling pathway, thereby affecting plaque stability [138]. Aβ causes vascular damage in the brain and OCT measurements show that the thickness of the retinal neuron and vascular layers of the eye are significantly thinner in AD patients [139,140]. Aβ brain deposition was positively correlated with vascular density in all regions of the retina [141]. One possible mechanism is the retrograde deformation of the optic nerve. Aβ pathological features visible in the subcortical visual centers could support this claim [142]. However, the combined approach of OCT and miRNA profiling in AD is rarely reported and possesses great potential for development.

### 7.3. Surface Plasmon Resonance (SPR)

Surface plasmon resonance can track biomolecular interactions in natural situations without any markers. The interaction processes between various biomolecules, such as peptides, proteins, oligonucleotides, and oligosaccharides, as well as viruses, bacteria, cells, and small molecule compounds, can be measured in real-time, in situ, and dynamically when passing through the sensor chip. The principle is that a biomolecule target is bonded to the biosensor surface, and then a solution containing an analyte that interacts with the target molecule is injected into and flows over the biosensor surface. The bonding between the biomolecules causes the refractive index to increase in the same proportion, and changes in the reaction between the biomolecules are observed. Song et al. [143] reported a DNA-assembled advanced plasma structure (DAPA)-based plasma biosensor capable of the selective single-nucleotide detection of exosomal miRNA at the attomolar level for AD diagnosis with an accuracy of 97.83%. The enhanced SPR biosensor enabled the high-throughput and ultra-sensitive screening of miRNAs and the simultaneous detection of multiple targets, which has great potential for future clinical diagnostics [144].

## 8. Outstanding Challenges for the Future

The multifaceted modulation of synapses by various miRNAs contributes to AD-related cognitive impairment. This review summarized key examples of miRNAs identified in recent years as upstream or downstream regulators of Aβ and tau proteins that modulate synaptic composition (synaptic vesicles and neurotransmitters), synaptic morphology, and synaptic function. Nevertheless, the research to date on miRNAs in AD has unresolved problems. Does the change in miRNA levels directly or indirectly contribute to synaptosome and synaptic function alterations and the toxicity induced by Aβ and tau proteins? Is the modulation of synapses by miRNAs the sole contributor to AD-related cognitive impairment? If not, how do we discover and verify the presence of other factors involved as well? Can the outcomes of miRNA expression-level abnormalities in situ at specific sites of pathogenesis be justified for commonly available humoral biomarkers (e.g., CSF and peripheral blood)? In response to the above questions, we need to consider whether the methodology for exploring somatic biomarkers is compatible with investigating the role of miRNAs in disease mechanisms. There has been considerable preclinical, basic research conducted on miRNAs in AD, but translational applications are still lacking.

## 9. Conclusions

In the brain, various cell types interact and functionally depend on each other in a tightly regulated manner. In particular, healthy vascular cells ensure sufficient cerebral blood flow and the release of trophic factors to nourish neurons. Aged vascular cells stimulate astrocytes to secrete inflammatory factors and damage synapses and neurons in pathological situations, such as AD. Perivascular immune cells (microglia and macrophages) and BBB play a monitoring role to investigate the molecules entering the brain. We presented a review of how miRNAs play an essential and role in the cellular interaction previously described, and how miRNA dysregulation affects AD-related cognitive impairment.

## Figures and Tables

**Table 1:** Summary of studies implying miRNA in synapse-associated phenotypes.

Phenotype	miRNA	Target Gene	References
Synaptic density	miR-135a-5p	ROCK2	[64]
	miR-138	SIRT1	[65]
Synaptic transmission	miR-26a-5p	-	[14]
	miR-135a-5p	ROCK2	[64]
	miR-484	-	[66]
Synaptic plasticity	miR-484	-	[66]
	miR-129	CAMK2D	[67]
		CAMK4	
		PPP3CA	
		PRKCB	
	miR-181a	GluA2	[51]
	miR-132	HDAC	[68]
		RBFOX1	[69]
	miR-135a-5p	ROCK2	[64]
	miR-124	PTPN1	[62]
Synaptotoxicity	miR-181a	GluA2	[51]
	miR-132	HDAC	[68]
Synaptic loss	miR-146a	NKD2	[27]
	miR-9-5p	SIRT1	[70]
Synaptic integrity	miR-30b	EPHB2	[71]
		SIRT1	
		GluA2	
	miR-34c	SYT1	[72]

## Data Availability

Not applicable.
